# Caprine arthritis and encephalitis virus infection in goats of Bangladesh: Serological detection and its associated risk factors

**DOI:** 10.14202/vetworld.2023.2256-2262

**Published:** 2023-11-11

**Authors:** Md. Habibur Rahman, Sonia Akther, Md. Shahin Alam, Md. Zulfekar Ali, Sadek Ahmed

**Affiliations:** 1 Goat Production Research Division, Bangladesh Livestock Research Institute, Savar, Dhaka 1341, Bangladesh; 2 Faridpur Regional Station, Bangladesh Livestock Research Institute, Faridpur, Bangladesh; 3 Sheep Production Research Division, Bangladesh Livestock Research Institute, Savar, Dhaka 1341, Bangladesh; 4 Animal Health Research Division, Bangladesh Livestock Research Institute, Savar, Dhaka 1341, Bangladesh; 5 Black Bengal Goat Conservation and Development Research Project, Bangladesh Livestock Research Institute, Savar, Dhaka 1341, Bangladesh

**Keywords:** Bangladesh, caprine arthritis and encephalitis, enzyme-linked immunosorbent assay, goat, risk factors, seroprevalence

## Abstract

**Background and Aim:**

Caprine arthritis and encephalitis (CAE) is a multisystemic persistent viral disease of goat that causes significant economic losses to the farmers and livestock sector. However, no information in this country is available regarding CAE virus (CAEV) infection. Therefore, this study aimed to estimate the seroprevalence of CAEV infection among the goat population in the selected goat-prone districts in Bangladesh and to identify the associated risk factors of the disease.

**Materials and Methods:**

From July 2021 to June 2022, 446 goat serum samples were randomly collected from the study area. Goat owners were interviewed using a pretested questionnaire to determine the risk factors. A commercial indirect enzyme-linked immunosorbent assay kit was used to screen blood serum for CAEV antibodies. Logistic regression models were used to analyze risk factors and serological data to identify the potential risk factors.

**Results:**

Out of 446 serum samples, 19 samples were seropositive against CAEV. The overall seroprevalence was 4.26% (95% confidence interval [CI]: 2.58–6.57). The multivariable logistic regression model identified sex (Female; odds ratio [OR]: 3.98; 95% CI: 1.13–13.95), animal age (12–48 months; OR: 4.93, 95% CI: 0.63–38.13), and biosecurity status (Poor biosecurity; OR: 1.66, 95% CI: 0.46–5.92) as potential risk factors for CAEV seropositivity.

**Conclusion:**

This study revealed the serological detection of CAEV in Bangladeshi goats where seroprevalence is found to be relatively low. To eradicate the disease, screening and culling of infected goats from the herd might be implemented.

## Introduction

Caprine arthritis and encephalitis (CAE) is a widespread, severe and fatal viral disease of goats caused by the CAE virus (CAEV), a lentivirus belonging to the retroviridae family [1–4]. It causes multisystemic inflammatory disease in small ruminants [5]. Lentivirus infections cause chronic, progressive, and devastating infections in various target organs including mammary gland, carpal joints, central nervous system, and lungs [3, 5]. This might have impacted the amount of milk produced and raised the possibility of developing mastitis [4, 6, 7]. The disease manifests as encephalitis in goat kids and severe arthritis, indurative mastitis, and occasionally interstitial pneumonia in adult goats [1, 8]. Additional clinical signs of CAEV infections, including joint enlargement leading to lameness, synovitis, and reduced growth rate, have been reported [2, 9, 10]. The arthritic form is more prevalent in goats, causing lameness, and increasing the diameter of the joints [11]. The disease’s economic losses result from mortality related to clinical illness, the low value of cull animals, the impact of subclinical disease on production, and, consequently, a decline in economic life [4, 12].

Normally, CAE virus infection transmission has been observed vertically and horizontally [2, 3, 12]. Vertical transmission occurs through ingestion of infected milk or colostrum’s and horizontal transmission occurs due to close contact with sick animals, body fluids, and excretions [2, 8]. However, unlike other Lentiviruses, CAEV’s sexual transmission has not yet been well explained [1]. The host is permanently infected once the virus enters into the body. There have also been reports of interspecies transmission occurring naturally within mixed populations, specifically goats to sheep and sheep to goats [3, 13]. CAE infection is prevalent throughout the world [2, 14]. The disease diagnosis process combines clinical manifestations, postmortem findings, and histopathological observations [2]. The detection of the maedi-visna (MV)/CAEV virus relies primarily on serological tests because the persistence of circulating antibodies against the virus is considered. These serological tests include agar gel immunodiffusion (AGID), enzyme-linked immunosorbent assay (ELISA), and indirect immunofluorescence which are used for serological detection of CAEV [1, 12]. In practical applications, the ELISA is the most commonly used serological test for diagnosing CAEV infection. Enzyme-linked immunosorbent assay is the preferred choice due to its higher sensitivity compared to AGID [1, 2].

At present, no available treatment or efficient vaccine for the disease has been developed but there remains a potential to eliminate it through enhancements in the quality and efficacy of diagnostic tests [12]. Hence, timely identification of the disease through serological test continues to be crucial for preventing, managing, and eradicating CAEV infection [15]. CAE virus infection has been reported in many countries since its first documentation in 1974 in goats [1, 16]. The distribution of CAEV is diverse, with significant differences between continents and sometimes within a single continent [17]. Therefore, the prevalence rate varied from country to country and has been recorded from Malaysia 8.8% [2], Brazil 6.2% and 8.2% [6, 7], Jordan 8.9% and 18.5% [14, 18], Thailand 5.52% [19], Oman 5.1 % [20], Turkey 7.5% [21], Mexico 3.6% [22], Pakistan 3.87% [23], Iraq 8.69% [24], Northern Somalia 6.0% [25], and Italy 18.64% [26]. Our neighboring country, India, also has seropositivity toward CAEV, with prevalence rates of 4.5% [5], 3.33% [10], and 6.96% [27]. However, there is no information available in this country regarding CAEV infection. The findings of this study will help the veterinary authorities in their future surveillance and disease control decisions.

This study aimed to estimate the seroprevalence of CAEV infection among goat population in selected goat-prone districts in Bangladesh and to identify the associated disease risk factors.

## Materials and Methods

### Ethical approval

The Animal Experimentation Ethics Committee of Bangladesh Livestock Research Institute (BLRI) approved this research project (Reference no.: AEEC/BLRI00110/2023). During sample collection, all the guidelines for animal care were carefully followed.

### Study period and location

The study was conducted from July 2021 to June 2022 in some selected goat-prone districts in Bangladesh, namely, Jashore, Jhenaidah, Chuadanga, Meherpur, Kustia, Mymensingh, Gaibandha, Dhaka, Rajshahi, and Bandarban.

### Sample size

**Figure-1:**
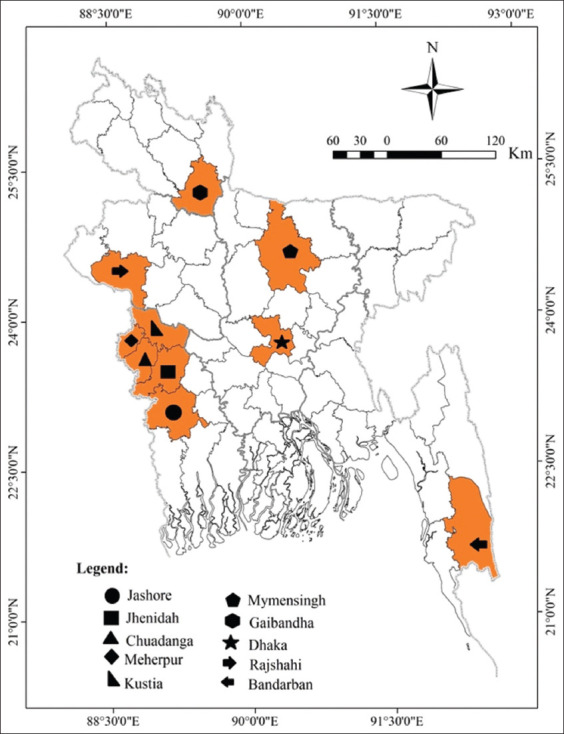
Map of Bangladesh depicting sampling location [Source: The map was generated by using ESRI ArcGIS version 10.6.1].

In these study areas, a total of 446 blood samples were collected from goats using a random sampling method, i.e., Jashore (n = 42), Jhenaidah (n = 36), Chuadanga (n = 52), Meherpur (n = 48), Kustia (n = 47), Gaibandha (n = 58), Rajshahi (n = 47), Mymensingh (n = 43), Dhaka (n = 41), and Bandarban (n = 32), as shown in Figure-1. Samples were collected from male and female Black Bengal goat (BBG), Jamunapari (JP), and crossbred goats for the investigation. After that, to identify the potential risk factors, some information about goat rearing was gathered from goat owners through direct interviews using a questionnaire [26].

Sample size (446 goats) was determined using a formula given by Thrusfield [28].

n = 1.96^2^P_exp_ (1–P_exp_)/d^2^

where,

n = required sample size

P_exp_ = expected prevalence

d = desired absolute precision (5%).

Since no previous study was found on the prevalence of CAEV in Bangladesh, the sample size was determined for a condition with an expected prevalence of 50% at a 95% confidence interval (CI) and a targeted absolute precision of 5% [28]. Hence, the total number of samples needed for this study was 384 goats; however, to obtain more accurate results, 446 goats were used as samples for investigation.

### Vaccination and rearing methods

All animals were Peste des Petits Ruminant vaccinated. All goats were raised semi-intensively and free-range rearing systems in the study area.

### Sample collection

Keeping the animals in a standing position and properly restrained by their owners, all blood samples were aseptically drawn from the jugular vein. A disposable syringe was used to draw 5 mL of blood from the jugular vein of each animal. The syringes were kept in an upright position at 27°C for approximately 2 h. The separated serum was quickly transported to the Small Ruminants Research Laboratory at the BLRI, Savar, Dhaka, in a 2 mL pre-labeled Eppendorf tube maintaining an appropriate cool chain. After that, all the collected serum was stored at −20°C until ELISA testing.

### Serological test

All collected sera were tested for anti-CAEV antibodies in the Small Ruminant Research Laboratory, BLRI, using a commercial ELISA kit (ID Screen® MVV/CAEV Indirect, ID vet, France, Batch: J39) following with the manufacturer’s instructions. The OD value was read at 450 nm by a Microplate reader (Thermo Scientific™ Multiskan™ FC Microplate Photometer, Thermo Fisher Scientific, USA). The samples with a percentage of inhibition (PI) ≥60% were classified as positive for the CAEV. The sensitivity and specificity rate of the kit are 99.3% and 99.7%, respectively, for the detection of CAEV antibody in the small ruminants [29].

### Risk factors

Breed, age, sex, rearing system, flock size, biosecurity status, and housing systems are the variables that were evaluated to determine their association with the seroprevalence of CAEV in goats. Results became statistically significant when p ≤ 0.05 in a 95% CI.

### Statistical analysis

A pretested questionnaire was applied to collect animal-level data from selected areas and data were entered, cleaned, coded, and checked in Microsoft Excel 2016 (Microsoft Corporation, Washington, USA) spreadsheet. Further, all epidemiological analyses were performed using STATA-13 (StataCrop, USA). The descriptive statistics were calculated to express the association of CAEV seropositivity to different variables, including goat breed, age, sex, rearing system, flock size, biosecurity status, housing system, and location. The univariate logistic regression analysis was performed to investigate the associations between risk factors (recoded into categorical variables) and CAEV seropositive results. Then, a multivariable logistic regression model was applied to identify the potential risk factors considering the variables with p < 0.20 in univariate analysis. The results for each predictor variable are shown as odds ratio (OR) and 95% CI. The seropositivity-related explanatory factors were identified using a backward stepwise approach.

## Results

### Descriptive statistics

The total seroprevalence of CAEV in goats was 4.26% (95% CI: 2.58–6.57), according to the indirect ELISA (iELISA) test against CAEV performed on 446 goats. In this study, the highest seropositivity rate was recorded in goats at Chuadanga with a prevalence of 7.69% (95% CI: 2.13–18.53) and the lowest seropositivity rate was recorded in Rajshahi with a prevalence of 2.13% (95% CI: 0.42–11.90), but no antibodies were detected in Bandarban. The distribution of seropositivity to CAEV among the ten districts is shown in Table-1. Statistically, there were no significant differences (p ≥ 0.05) between districts.

**Table-1:** District-wise seroprevalence of caprine arthritis encephalitis virus in goats tested by iELISA.

Sampling location	No. of sera tested	Positive	Prevalence (%)	95% CI	OR (95% CI)	p-value
Jashore	42	2	4.76	0.58–16.16	2.3 (0.20–26.33)	0.503
Jhenaidah	36	2	5.55	0.68–18.66	2.7 (0.23–31.07)	0.424
Chuadanga	52	4	7.69	2.13–18.53	3.83 (0.41–35.58)	0.237
Meherpur	48	3	6.25	1.30–17.19	3.06 (0.30–30.59)	0.34
Kustia	47	2	4.25	0.51–14.54	2.04 (0.17–23.34)	0.565
Gaibandha	58	2	3.45	0.42–11.90	1.64 (0.14–18.69)	0.689
Rajshahi	47	1	2.13	0.05–11.29	1.0	-
Mymensingh	43	2	4.65	0.56–15.81	2.24 (0.19–25.66)	0.516
Dhaka	41	1	2.44	0.06–12.85	1.15 (0.06–18.98)	0.922
Bandarban	32	0	-	-	-	-
Total	446	19	4.26	2.58–6.57		

*CI=Confidence interval, OR=Odds ratio, iELISA=Indirect enzyme-linked immunosorbent assay

### Univariate analysis

Considering breeds of goat, the highest seroprevalence was found in crossbred goats (4.8%; 95% CI: 2.44–8.5) compared to Black Bengal (4.20%; 95% CI: 1.55–8.89) and Jamunapari goats (2.63%; 95% CI: 0.32–9.18), but no significant differences were found. There was a statistically significant difference in the seropositivity recorded between age groups (p < 0.05); adults (5.86%, 95% CI: 3.38–9.34, OR: 4.1) were more prone to CAEV infection as compared to young (<12 month). There was also a statistically significant difference in the seropositivity among sexes of goats (p < 0.05) in which female goats were more affected. In addition, higher seroprevalence was found in the free-ranging rearing system (4.58%, 95% CI: 2.64–7.33, OR: 1.5) compared to the semi-intensive rearing system. The flock sizes, that is, small, medium, and large showed 4.43%, 4.76%, and 3.71% seropositivity, respectively. In case of biosecurity of goat farms, having poor biosecurity (4.58%, 95% CI: 2.64–7.33, OR: 1.5) compared to the good biosecurity status were found significantly more prone to seropositivity. In housing system, seropositivity of CAEV in goats was higher in the goats reared in the floor system (4.58%, 95% CI: 2.64–7.33, OR: 1.5) compared to the slat system and the relation was non-significant (Table-2).

**Table-2:** Univariable logistic regression analysis of risk factors for caprine arthritis encephalitis virus seropositivity of goats in study area.

Variable	Category	No. of tested (% positive)	95% CI	OR (95% CI)	p-value
Breed	Black Bengal goat	143 (4.20)	1.55–8.89	1.62 (0.32–8.23)	0.122
	Crossbreed	227 (4.80)	2.44–8.5	1.88 (0.40–8.69)	0.847
	Jamunapari	76 (2.63)	0.32–9.18	1.0	-
Age (month)	<12	106 (1.89)	0.02–6.64	1.26 (0.11–14.27)	0.057
	12–48	273 (5.86)	3.38–9.34	4.1 (0.53–31.54)	0.046
	>48	67 (1.49)	0.03–8.03	1.0	-
Sex	Male	179 (1.68)	0.34–4.81	1.0	-
	Female	267 (5.99)	3.46–9.54	2.82 (0.80–9.88)	0.038
Rearing system	Free	349 (4.58)	2.64–7.33	1.5 (0.42–5.27)	0.523
	Semi	97 (3.09)	0.64–8.77	1.0	-
Flock size	Small	316 (4.43)	2.44–7.32	1.31 (0.36–4.67)	0.674
	Medium	42 (4.76)	0.5–16.16	1.41 (0.22–8.81)	0.709
	Large	88 (3.41)	0.70–9.64	1.0	-
Biosecurity status	Good	97 (3.09)	0.64–8.77	1.0	-
	Poor	349 (4.58)	2.64–7.33	1.5 (0.42–5.27)	0.062
Housing system	Floor	349 (4.58)	2.64–7.33	1.5 (0.42–5.27)	0.523
	Slat	97 (3.09)	0.64–8.77	1.0	-

CI=Confidence interval, OR=Odds ratio

### Multivariate analysis

In multivariable logistic regression analysis, three factors (variables) were predicted as potential risk factors for the seroprevalence of CAEV in goats of the selected location by adapting the result of the factors with each other, including age, sex and biosecurity status of goat farms. Goats aged between 12 and 48 months (OR: 4.93, 95% CI: 0.63–38.13), female sex (OR: 3.98, 95% CI: 1.13–13.95), and goat farmers with poor biosecurity status (OR: 1.66, 95% CI: 0.46–5.92) were found to be more likely to acquire the seropositive against CAEV (Table-3).

**Table-3:** Results of multivariable logistic regression analysis of potential risk factors associated with caprine arthritis encephalitis virus seropositivity of goats.

Variable	Category	Adjusted OR (95% CI)	p-value
Age (month)	<12	1.44 (0.12–16.33)	0.067
	12–48	4.93 (0.63–38.13)	0.012
	>48	1.0	-
Sex	Male	1.0	-
	Female	3.98 (1.13–13.95)	0.031
Biosecurity status	Good	1.0	-
	Poor	1.66 (0.46–5.92)	0.043

CI=Confidence interval, OR=Odds ratio

## Discussion

Caprine arthritis and encephalitis is a viral disease that affects goats, causing persistent fatigue and significant economic impact. The disease primarily presents as subclinical, although a minority of the animals in the goat population may exhibit certain signs and symptoms [1]. Prolonged pneumonia, neurological disorders, arthritis, and chronic mastitis have been identified as prevalent illnesses in goats with infection by CAEV becoming the main cause. These conditions significantly contribute to reduced productivity and reproductive capabilities, thereby increasing morbidity and mortality rates in affected goats [2].

In Bangladesh, there are no available data on CAEV infection in goats. This is the first epidemiological investigation of CAEV disease in Bangladeshi goats. Goats with high infection rates have lower lifetime production, and additionally, prevent goat exports from endemic countries [2, 20]. Rahman *et al*. [30] stated that 9% of goats in Bangladesh have lameness caused by arthritis and some mechanical causes. Caprine arthritis and encephalitis virus receive no attention and typically goes undetected or incorrectly diagnosed in veterinary hospitals. The results of this investigation provide information regarding the serological identification of CAEV infection. Using iELISA, the overall seroprevalence was found 4.26% in Bangladeshi goats. Almost similar seroprevalence was recorded in Andhra Pradesh, India (4.5%), according to Didugu *et al*. [5] and Kenya (4.5%), according to Adams *et al*. [31]. Although our finding is about 4 times lower than Jordan’s 18.5% [18] and Italy’s 18.64% [26], about 2 times lower than Malaysia 8.8% [2], Brazil 8.2% [7], Turkey 7.5% [21], and Iraq 8.69% [24]. On the other hand, it is higher than India 3.33% [10], Mexico 3.6% [22], Pakistan 3.87% [23], and Turkey 1.35% [32]. The prevalence of CAEV could be different in different places due to climate, livestock management practices, and housing conditions, as these variables have been mentioned in the occurrence of infectious diseases in the past [33, 34]. Furthermore, prior studies are not comparable with the present study due to variations in the sample number, sampling time, sampling location, disease susceptibility of different breeds, animal management procedures, applying different tests, and different sample analysis principles.

According to Jesse *et al*. [2] and Peterhans *et al*. [16] herd management, goat breed, flock size, and animal age are the risk factors for higher CAEV occurrence in several countries. This aligns with the findings of the present study, which found that CAEV seropositivity is higher in crossbred (4.80%), goats aged 12–48 months (5.86%), and farms with poor biosecurity (4.58%). In this investigation, CAEV seroprevalence was significantly associated with sex. Female goats (5.99%) were more CAEV-positive than males (1.68%). This result is consistent with the findings of Jesse *et al*. [2], Waseem *et al*. [10], and Norouzi *et al*. [12], but, contrary to the findings of Bandeira *et al*. [7], who found higher seropositivity in bucks compared to does. The variation in results could be explained by the number of goats sampled, their sexes, and the types of tests performed.

Our study showed a statistically significant increase in CAEV seropositivity among animals between 12 and 48 months of age (5.86%) than other age groups. Similarly, Alamerew *et al*. [1], Jesse *et al*. [2], Norouzi *et al*. [12], and Hamzah and Mosa [24] found that older goats were more CAEV seropositive than younger goats. The lower seropositivity observed in younger individuals can potentially be explained by the humoral immunity of the animal. The best explanation for an increase in prevalence through age is horizontal transmission carried on by interaction with goats affected by CAEV. As CAEV is chronic and can cause lifelong infection in hosts, older animals with a higher chance of exposure to the various risk factors have a greater chance of being at risk, becoming infected, and remaining sick [1]. However, Wassem *et al*. [10] reported that CAEV infects goats of any age, breed, and sex.

In this study, crossbred goats had higher seropositivity than black Bengal and Jamunapari goats, due to the larger number of samples were taken from this breed. However, there was no significant relationship between goat breed and CAEV seroprevalence. This result is consistent with the findings of Jesse *et al*. [2], who reported no significant relationship between breed and CAEV infection. Because the disease could be transmitted between species, sheep need to be included in the prevention strategies [17, 20]. Goats and sheep are raised together in Bangladesh. Therefore, chance of interspecies transmission is possible. However, the investigation of lentivirus (MVV) in sheep is recommended. The results of our study showed that small (1–6 goats) and large (> 40 goats) flocks had lower seroprevalence of CAEV than the medium-sized (7–40 goats) flocks, though this difference was not statistically significant. This finding conflicts with that of Al-Qudah *et al*. [14], who predicted that high stocking densities and large flock sizes were risk factors. In Bangladesh, a significant proportion of rural goat farmers typically maintain a flock size ranging from 5 to 40 goats. Hence, it is predicted that the prevalence of the disease will be higher in flocks of this particular size. However, Cutlip *et al*. [35] proposed that the size of a herd has no effect on seropositivity to CAEV. We found goats reared by a free-ranging system with Kacha floor (native Bengali word), not using slat system housing also had a higher seroprevalence of CAEV than goats reared by a semi-intensive system with a slat type housing. This may be due to, most of the farmers in our study area reared their goats by a free-ranging system and not using slat system housing. However, the free movement of animals and the greater number of samples collected from these farmers also contribute to the higher seroprevalence of CAEV. However, Al-Qudah *et al*. [14] stated that the rearing method does not make a big difference in CAEV seropositivity.

In this study, farms with poor biosecurity management had higher seropositivity than those with good biosecurity. Al-Qudah *et al*. [14] and Potârniche *et al*. [36] found that poor biosecurity and sanitation practices increase CAEV spread. Biosecurity-managed farms have limited external exposure so lower seroprevalence was observed on these farms. The study used only serological test within some limited areas. A national epidemiological investigation using PCR or other highly accurate diagnostic assays is recommended to assess the disease’s prevalence in small ruminants.

## Conclusion

This study showed that CAEV infection exists in Bangladeshi goat flocks but does not appear to be prevalent. The overall seroprevalence of CAE was 4.26%. Seropositivity differed significantly depending on the goat’s sex, age group, and biosecurity status. To reduce CAE’s major financial problems in the country’s livestock industry and its public health consequences, it is important to perform diagnostic tests periodically to comprehend the disease’s progression. A nationwide epidemiological research and molecular analysis are recommended to gain an in-depth knowledge of CAE, particularly its root causes and origins.

## Authors’ Contributions

MHR: Conceptualization, data and sample collection, laboratory test, data analysis and interpretation, and drafted and revised the manuscript. SoA: Laboratory test and reviewed the manuscript. MSA: Designed the study, supervised, and reviewed and edited the manuscript. MZA: Data analysis and interpretation, revised the manuscript, and critical review. SA: Supervised and reviewed and edited the manuscript. All authors have read, reviewed, and approved the final manuscript.

## References

[1] Alamerew E.A, Demis C, Asfaw T, Gemeda B.A, Asres F.A, Yitagesu E, Wondifra Y, Areaya A (2022). Serological evidence of caprine arthritis encephalitis in North Shewa Zone, Ethiopia:Clinical case analysis. Vet. Med. (Auckl).

[2] Jesse F.F, Bitrus A.A, Abba Y, Raju V.N, Hambali I.U, Peter I.D, Lila M.A, Norsidin J.M (2018). Seroprevalence of small ruminant caprine arthritis encephalitis lentivirus among goats from selected small ruminant farms in Selangor, Malaysia. Vet. World.

[3] Thomann B, Falzon L.C, Bertoni G, Vogt H.R, Schüpbach-Regula G, Magouras I (2017). A census to determine the prevalence and risk factors for caprine arthritis-encephalitis virus and visna/maedi virus in the Swiss goat population. Prev. Vet. Med.

[4] Peterson K, van den Brom R, Aalberts M, Bogt-Kappert C.T, Vellema P (2022). Loss of caprine arthritis encephalitis virus (CAEV) Herd accreditation:Characteristics, diagnostic approach, and specific follow-up scenarios on large dairy goat farms. Pathogens.

[5] Didugu H, Sagi S, Reddy C.E, Kishore K.N, Reddy M.V, Vishnu P.G (2016). First report of maedi-visna and caprine arthritis-encephalitis viruses in Krishna district, Andhra Pradesh. Indian J. Anim. Res.

[6] De Sousa M.M, Andrioli A, Pinheiro R.R, Alves F.S.F, Dos Santos V.W.S (2019). An epidemiological study of caprine arthritis encephalitis virus (CAEV) in breeder goats from Northeastern Brazil [Estudo epidemiológico da Artrite Encefalite Caprina ávírus (CAEV) com ênfase em reprodutores de rebanhos do Nordeste do Brasil]. Semin. Ciênc. Agrár.

[7] Bandeira D.A, de Castro R.S, Azevedo E.O, Melo L.S.S, De Melo C.B (2009). Seroprevalence of Caprine arthritis-encephalitis virus in goats in the Cariri region, Paraiba state, Brazil. Vet. J.

[8] Mosa A.H, Hamzah K.J, Aljabory H.A (2022). First study on the molecular prevalence of caprine arthritis encephalitis virus in goats in Babylon, Iraq. Vet. World.

[9] Gomez-Lucia E, Barquero N, Domenech A (2018). Maedi-Visna virus:Current perspectives. Vet. Med. (Auckl).

[10] Waseem A, Pawaiya R.V, Singh R, Gupta V.K, Rajukumar K, Mir M.S, Aamir S (2015). Seroprevalence of caprine arthritis encephalitis virus infection (CAEV) in Indian goats. Indian J. Vet. Pathol.

[11] De Souza T.S, Pinheiro R.R, Costa J.N, de Lima C.C, Andrioli A, de Azevedo D.A, dos Santos V.W, Araújo J.F, Sousa A.L, Pinheiro D.N, Fernandes F (2015). Interspecific transmission of small ruminant lentiviruses from goats to sheep. Braz. J. Microbiol.

[12] Norouzi B, Razavizadeh A.T, Azizzadeh M, Mayameei A, Mashhadi V.N (2015). Serological study of small ruminant lentiviruses in sheep population of Khorasan-e-Razavi province in Iran. Vet. Res. Forum.

[13] Gjerset B, Rimstad E, Teige J, Soetaert K, Jonassen C.M (2009). Impact of natural sheep-goat transmission on detection and control of small ruminant lentivirus group C infections. Vet. Microbiol.

[14] Al-Qudah K, Al-Majali A.M, Ismail Z.B (2006). Epidemiological studies on caprine arthritis-encephalitis virus infection in Jordan. Small Rumin. Res.

[15] Brinkhof J.M.A, Moll L, Van Maanen C, Houwers D.J (2010). Use of serology and polymerase chain reaction for the rapid eradication of small ruminant lentivirus infections from a sheep flock:A case report. Res. Vet. Sci.

[16] Peterhans E, Greenland T, Badiola J, Harkiss G, Bertoni G, Amorena B, Eliaszewicz M, Juste R, Krassnig R, Lafont J.P, Lenihan P (2004). Routes of transmission and consequences of small ruminant lentiviruses (SRLVs) infection and eradication schemes. Vet. Res.

[17] De Miguel R, Arrieta M, Rodríguez-Largo A, Echeverría I, Resendiz R, Pérez E, Ruiz H, Pérez M, de Andrés D, Reina R, de Blas I (2021). Worldwide prevalence of small ruminant lentiviruses in sheep:A systematic review and meta-analysis. Animals (Basel).

[18] Hailat N.Q, Algharaibeh T.B, Al-Eitan L.N (2022). Pathological, molecular, and serological study of small ruminant lentiviruses in Jordan. Vet. World.

[19] Nyi Lin T, Ngarmkum S, Oraveerakul K, Virakul P, Techakumphu M (2011). Seroprevalence and risk factors associated with caprine arthritis-encephalitis virus infection in goats in the western part of Thailand. Thai. J. Vet. Med.

[20] Tageldin M.H, Johnson E.H, Al-Busaidi R.M, Al-Habsi K.R, Al-Habsi S.S (2012). Serological evidence of caprine arthritis-encephalitis virus (CAEV) infection in indigenous goats in the Sultanate of Oman. Trop. Anim. Health Prod.

[21] Azkur A.K, Gazyagci S, Aslan M.E (2011). Serological and epidemiological investigation of bluetongue, Maedi-Visna and caprinearthritis-encephalitis viruses in small ruminant in Kirikkale District in Turkey. Kafkas Univ. Vet. Fak. Derg.

[22] Torres-Acosta J.F, Gutierrez-Ruiz E.J, Butler V, Schmidt A, Evans J, Babington J, Bearman K, Fordham T, Brownlie T, Schroer S, Cámara G.E (2003). Serological survey of caprine arthritis-encephalitis virus in 83 goat herds of Yucatan, Mexico. Small Rumin. Res.

[23] Mahmood F, Khan A, Khan M.Z, Hussain R, Gul S.T, Siddique A.B (2012). Pathological and molecular based study of naturally occurring lentivirus infection. Pak. Vet. J.

[24] Hamzah K.J, Mosa A.H (2020). Clinical and serological diagnosis of caprine arthritis encephalitis virus (CAEV) in goats of middle Iraq regions. Life Sci. Arch.

[25] Ghanem Y.M, EL-Khodery S.A, Saad A.A, Elragaby S.A, Abdelkader A.H, Heybe A (2009). Prevalence and risk factors of caprine arthritis encephalitis virus infection (CAEV) in Northern Somalia. Small Rumin. Res.

[26] Cirone F, Maggiolino A, Cirilli M, Sposato A, De Palo P, Ciappetta G, Pratelli A (2019). Small ruminant lentiviruses in goats in southern Italy:Serological evidence, risk factors and implementation of control programs. Vet. Microbiol.

[27] Singh R, Singh R, Kumari S (2018). Seroprevalence study of small ruminant lentivirus infection in Indian sheep and goats. J. Entomol. Zool. Stud.

[28] Thrusfield M (2018). Veterinary Epidemiology. John Wiley and Sons, Ltd., Chichester, UK.

[29] Jerre A, Nordstoga A.B, Dean K.R, Holmøy I.H (2022). Evaluation of three commercial ELISA tests for serological detection of maedi-visna virus using Bayesian latent class analysis. Prev. Vet. Med.

[30] Rahman M.H, Akther S, Ali M.Z, Hassan M.Z (2020). Incidence of diseases in goats in Bangladesh. Bangladesh Vet.

[31] Adams D.S, Oliver R.E, Ameghino E, DeMartini J.C, Verwoerd D.W, Houwers D.J, Waghela S, Gorham J.R, Hyllseth B, Dawson M, Trigo F.J, McGuire T.C (1984). Global survey of serological evidence of caprine arthritis encephalitis virus infection. Vet. Rec.

[32] Gumusova S.O, Memıs Y.S (2016). Caprine arthritis encephalitis and bluetongue virus infections in Maltese, Saanen and hair goat breeds. Pak. J. Zool.

[33] Ahmed A.G.M, Bakri E.O, Hussien M.O, Taseen M.E.E, Ahmed A.M, Abdalla M.A (2020). Molecular detection and risk factors of African horse sickness virus (ahsv) in different governorates of Sudan. J. Anim. Health Prod.

[34] Zarea Z.Z, El-Demerdash G.O, El-Shafei A.A, Abd Elkader S.A (2021). Occurrence of *Escherichia coli* as a causative agent of enteritis in dogs with special reference to their multidrug resistance and virulence genes. J. Anim. Health Prod.

[35] Cutlip R.C, Lehmkuhl H.D, Sacks J.M, Weaver A.L (1992). Prevalence of antibody to caprine arthritis-encephalitis virus in goats in the United States. J. Am. Vet. Med. Assoc.

[36] Potârniche A.V, Cerbu C, Olah D, Suatean M, Peredi C, Guranda S, Spînu M (2018). Serological survey of caprine arthritis-encephalitis virus infection in Sibiu county, Romania. Sci. Works Ser. C. Vet. Med.

